# Molecular and Nonmolecular Imaging of Macrophages in Atherosclerosis

**DOI:** 10.3389/fcvm.2021.670639

**Published:** 2021-05-19

**Authors:** Zhaoyue Li, Hao Tang, Yingfeng Tu

**Affiliations:** Department of Cardiology, First Affiliated Hospital of Harbin Medical University, Harbin, China

**Keywords:** macrophage, atherosclerosis, molecular imaging, multimodal imaging, optical coherence tomography

## Abstract

Atherosclerosis is a major cause of ischemic heart disease, and the increasing medical burden associated with atherosclerotic cardiovascular disease has become a major public health concern worldwide. Macrophages play an important role in all stages of the dynamic progress of atherosclerosis, from its initiation and lesion expansion increasing the vulnerability of plaques, to the formation of unstable plaques and clinical manifestations. Early imaging can identify patients at risk of coronary atherosclerotic disease and its complications, enabling preventive measures to be initiated. Recent advances in molecular imaging have involved the noninvasive and semi-quantitative targeted imaging of macrophages and their related molecules *in vivo*, which can detect atheroma earlier and more accurately than conventional imaging. Multimodal imaging integrates vascular structure, function, and molecular imaging technology to achieve multi-dimensional imaging, which can be used to comprehensively evaluate blood vessels and obtain clinical information based on anatomical structure and molecular level. At the same time, the rapid development of nonmolecular imaging technologies, such as intravascular imaging, which have the unique advantages of having intuitive accuracy and providing rich information to identify macrophage inflammation and inform targeted personalized treatment, has also been seen. In this review, we highlight recent methods and research hotspots in molecular and nonmolecular imaging of macrophages in atherosclerosis that have enormous potential for rapid clinical application.

## Introduction

**Figure 1:**
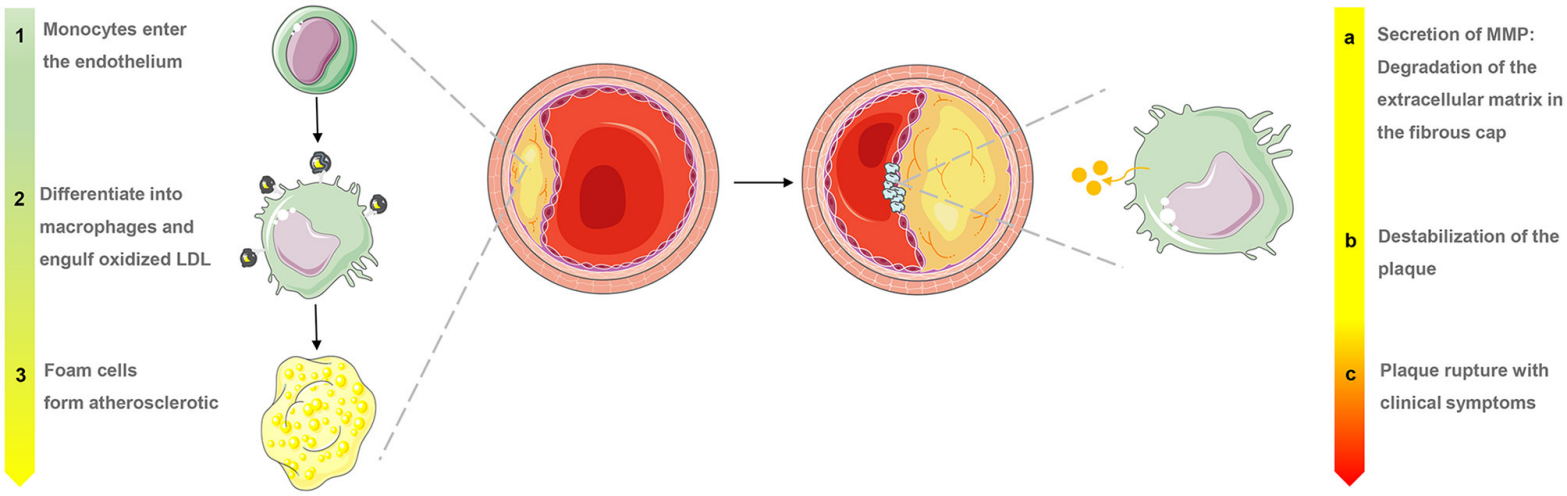
Macrophage evolution in progressive stages and the role of matrix metalloproteinases (MMP) in the late stages of atherosclerosis.

Atherosclerosis is a major cause of ischemic heart disease, and the increasing medical burden associated with atherosclerotic cardiovascular disease has become a major global public health concern (1, 2). Many factors have been linked to atherosclerosis, including the accumulation of inflammatory infiltration and immune cell activation. One of the first processes in the pathogenesis of atherogenesis is macrophage accumulation within the sub-endothelium or neointima constitutes, at which point scavenger receptors expressed by monocytes and macrophages take up lipoproteins and become lipid-loaded foam cells (3). During this process, macrophages continually secrete inflammatory cytokines and amplify the inflammatory response. However, macrophage proliferation may take on a more important role in advanced necrotic atherosclerotic lesions that exhibit a pattern of progression from pathologic intimal thickening to fibroatheroma with a lipid-rich necrotic core (Figure 1) (4, 5). There are no clinical signs or symptoms in the early stages of atherosclerosis, and ischemic symptoms do not appear until the atherosclerotic plaque has blocked or even occluded blood vessels (6). Macrophages play a significant role in all stages of the dynamic progression of atherosclerosis, from its initiation and lesion expansion increasing the vulnerability of plaques, to the formation of unstable plaques and clinical manifestations (7). Researchers have found that clinical imaging can detect the presence and activation of macrophages, which may help in the identification of patients who are at risk of coronary atherosclerotic disease and its complications, enabling preventive measures to be taken.

Multiple techniques have been used for macrophage imaging, including noninvasive imaging using nanoparticles designed according to the metabolic activity and phagocytosis characteristics of macrophages, and invasive imaging, which directly displays macrophages in atherosclerosis using high resolution (8, 9). Molecular imaging techniques are widely used in animal models as well as in the clinical setting; these include surface-enhanced Raman spectroscopy (SERS), bioluminescence imaging (BLI), near-infrared fluorescence (NIRF), laser scanning intravital microscopy (IVM), contrast-enhanced ultrasound (CEU), magnetic resonance imaging (MRI), positron emission tomography (PET), and single-photon emission computed tomography (SPECT). Multimodal imaging provides multi-dimensional imaging and the comprehensive assessment of blood vessels, offering more accurate information for the diagnosis of disease in comparison with the complementary capabilities of a single method. Interest in multimodal imaging has promoted the application of molecular imaging research in clinical diagnosis, providing clinical information on the occurrence and development of disease based on anatomical structure at the molecular level. In addition to molecular imaging, optical coherence tomography (OCT) and OCT-NIRF have been used to identify macrophages *in vivo*. OCT can qualitatively and quantitatively identify macrophages and determine the vulnerability of plaques based on the inflammatory infiltration. Moreover, intravascular OCT-NIRF can not only visually image the cellular-level anatomical structure of macrophages in atherosclerosis, but it can also simultaneously display molecular level information, such as enzyme activity.

In this review, we illuminate recent methods and research hotspots in molecular and nonmolecular imaging of macrophages in atherosclerosis that have enormous potential for rapid clinical transformation. Table 1 summarizes the resolution, characteristics, and advantages of each imaging modality.

**Table 1:** Summary of imaging modalities to identify macrophage in atherosclerosis.

**Method**	**Resolution**	**Strengths**	**Limitations**	Performance^*^	**Clinical use**
**Molecular imaging**					
SERS	1 μm	Label-free analysis, high spatial resolution, and highly detailed classification of tissue morphology	Low sensitivity can prolong imaging times, poor signal-to-noise in some tissues, require complex chemometric analysis to separate analytes	Cellular level	None yet
BLI	0.1–2 mm	Excellent sensitivity, no radiation, good temporal resolution, and multiplexing capability	Limited depth of penetration, poor spatial resolution at deeper tissue, and surface bioluminescence imaging	Animal level; *in vitro*	None yet
NIRF	1 μm−1 mm	Relatively low cost, no radiation, moderate multiplexing capability	Requires hybrid technologies for higher resolution imaging, relatively broad emission spectrum limits multiplexing, and the potential toxicity of imaging agents	Animal level; *in vivo*	None yet
IVM	1 μm	Cellular resolution, dynamic	Shallow penetration depth, invasive	Animal level; *in vivo*	None yet
CEU	50 μm	Low cost, no radiation, high speed, and sequential imaging, and amenable to bedside testing	Poor sensitivity and signal-to-noise ratio make molecular imaging challenging, lack of vascular penetration confines information to the endothelial surface	Animal level; *in vivo*	Plaque morphology, thrombus and ulceration detection, and stenosis severity
MRI	10 μm−1 mm	Excellent soft-tissue contrast for plaque characterization, non-ionization radiation	Poor sensitivity, long imaging times often required, and poor signal-to-noise ratio	Animal level; *in vivo*	Plaque inflammatory burden, morphology, and stenosis severity
PET/SPECT	1–5 mm	Unrestricted imaging depth, non-invasive	Poor spatial resolution, radiation exposure, requires CT integration for anatomical analysis/quantification	Animal level; *in vivo*	Plaque inflammatory burden
**Nonmolecular imaging**					
OCT	10 μm	High resolution of clinical techniques	High cost, invasive	Animal level/clinical use; *in vivo*	Plaque inflammatory burden, morphology, stenosis severity, and microarchitecture
OCT-NIRF	10 μm	Feedback plaque characteristics and cell and molecule metabolism at the same time	Low image acquisition rate, invasive	Animal level/clinical use; *in vivo*	Plaque inflammatory burden/activity, plaque morphology, stenosis severity, and microarchitecture

* *Performance refers to the ability to detect macrophages, and to image macrophages in vivo or in vitro. SERS, surface-enhanced Raman spectroscopy; BLI, bioluminescence imaging; NIRF, near-infrared fluorescence; IVM, laser scanning intravital microscopy; CEU, contrast-enhanced ultrasound; MRI, magnetic resonance imaging; PET, positron emission tomography; SPECT, single-photon emission computed tomography; OCT, optical coherence tomography; IVUS, intravascular ultrasonography*.

## Surface-Enhanced Raman Spectroscopy

Raman spectroscopy provides value in visualization at the single-cell level and can be applied for the detection of cell activation as well as metabolic events, without the need for additional fluorescent probes. Raman spectroscopy does not only provide structural information on intracellular molecules but also reveals the differences and dynamic changes of biochemical components between certain cells by detecting the vibration characteristics of multiple lipid classes (10, 11). Initially, Matthaus et al. (12) used Raman spectroscopy to study the lipid uptake dynamics of macrophages, providing a detection method for early atherosclerosis. In a subsequent study, the same group used isotope labeling combined with Raman imaging to investigate the dynamics of fatty acid storage in macrophages and found that this not only efficaciously tracked living macrophages, but also reflected macrophage lipid uptake through the collection of real-time signal fluctuation data (13).

**Figure 2:**
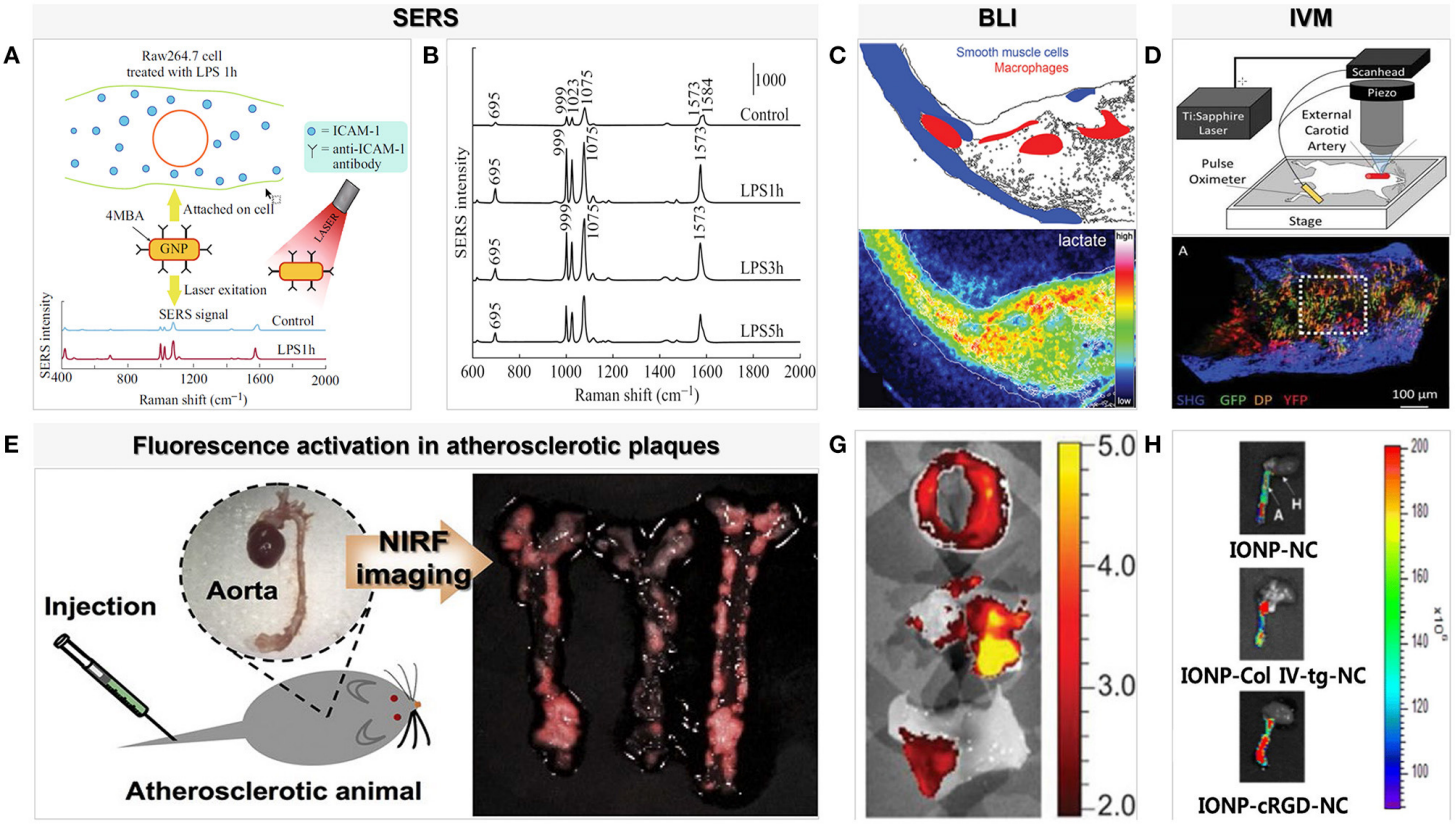
Examples of surface-enhanced Raman spectroscopy and optical imaging for macrophages in atherosclerosis. **(A)** SERS probe GNR techniques for detection of adhesion molecules expressed on the surface of macrophage cells (Raw264.7) [adapted from (14)]. **(B)** SERS spectra detected from Raw264.7 cells treated with LPS for different lengths of time (1, 3, and 5 h). The SERS spectra were averaged from ~80 to 235 spectra detected for each condition [adapted from (14)]. **(C)** Bioluminescence metabolic imaging showed energy metabolism in shoulder region of human atherosclerotic lesion in the common carotid artery [adapted from (16)]. **(D)** An aorta explanted from an Apoe^−/−^ Cx3cr1GFP/+CD11cYFP mouse fed WD and imaged with two-photon microscopy shows GFP, DP, and YFP cells in the wall [adapted from (17)]. **(E)** NIRF imaging of atherosclerotic plaques in ApoE KO mice. P-ICG2-PS-Lip was intravenously injected into ApoE KO mice via a tail vein, and the images were obtained by using a Maestro fluorescence imaging system [adapted from (18)]. **(G)** *Ex vivo* imaging with GB123 in human carotid plaque (above: unstable plaque + inhibitor, middle: unstable plaque, below: stable plaque) revealed high cathepsin activity, in yellow, was found in probe-treated unstable plaques [adapted from (19)]. **(H)** NIR fluorescence images of collected aorta from ApoE KO mice after 24 h injection of IONP-cRGD-NC, IONP-Col IV-tg-NC, IONP-NC [adapted from (20)].

Although the chemical information of molecules detected using Raman spectroscopy is limited, the signal is improved using surface-enhanced Raman scattering, which combines precious metal (gold or silver) nanoparticles. This is due to enhanced excitation and scattering of the plasmon when the molecule is adsorbed on or is close to the metal surface. On this basis, Pissuwan and Hattori (14) designed a surface-enhanced Raman scattering gold nanorod probe specifically for binding endothelial intercellular adhesion molecule-1 produced by macrophages and the part of cytokines that stimulates endothelial cells in order to enhance Raman signals and achieve better imaging results at the cellular level (Figure 2). A facile method of fabricating hollow-channel gold nanoflowers without surface activators for surface-enhanced Raman scattering was recently developed by the Ye et al. (15), and the trimodal nanoprobes demonstrated effective cellular internalization and low cell toxicity. The development of less toxic silver or gold nanoparticles with highly specific particles will be possible in the near future for use *in vivo* with emission profiles to allow in-depth analysis of tissues. Moreover, the nanoparticles were functionalized with targeting molecules and tuned to work across a wide range of wavelengths from the visible to the near-infrared to achieve accuracy and real-time *in vivo* diagnosis.

## Optical Imaging

### Bioluminescence Imaging

Based on the luciferase-mediated chemiluminescence detection of oxidation reactions, bioluminescence imaging (BLI) is a noninvasive technique for optical imaging that is commonly used in whole-body imaging of cell populations in small animal models (21). The first step of BLI in macrophage imaging involves *ex vivo* labeling utilizing a lentivirus vector that encodes given luciferase-encoding genes in the target macrophage. These engineered cells are then intravenously injected into the animal body, and luciferase enzymes expressed in engineered cells then catalyze light emissions during the luciferin oxidation reaction. The luciferase–luciferin system of BLI with green fluorescent protein (GFP) and firefly luciferase (FLUC) may be a powerful tool for studying macrophage biology. Pajarinen et al. used the double infection strategy in an attempt to solve the problem of low transfection efficiency from efficient gene transfer to primary macrophages. Firstly, they designed *ex vivo* labeling by using a lentivirus vector and cyclosporine to produce mouse primary macrophages with a strong expression of GFP/FLUC (up to 60%). The engineered cells were then transferred into the mouse model, and after a period of observation, they crowded in areas of chronic inflammatory activation (22). However, BLI is limited by tissue specificity, transfection efficiency, and bioluminescence duration. The new luciferase/luciferin systems and their related tools will promote the application of multicolor BLI for more information obtained in refined animal experiments. Moreover, BLI could be used to advance genetically modified animals by expanding the application of gene editing technology, rather than focusing on the transformation of research into human applications.

### Fluorescence Imaging and Near-Infrared Fluorescence

Fluorescence imaging is an alternative solution that can achieve long-term and whole-body macrophage tracking. The traditional method of fluorescence imaging may not easily reach the level of sensitivity required for clinical application, because of the rapid attenuation of photons in the detection process and the visible light signal being mostly absorbed by the *vivo* tissue. However, photon absorption by hemoglobin, lipids, and water in body tissues was avoided due to the volume of the probe being significantly smaller than that of the endogenous photon absorber. Nonetheless, when tissue autofluorescence imaging was minimized, especially in the near-infrared region, the signal was found to be significantly improved. NIRF imaging which can be coupled with activatable fluorescent probes targeting macrophages in the atherosclerotic lesion, can improve the accuracy of macrophage detection and serve as a tool for the detection of unstable atherosclerotic plaques (23–25).

It is noteworthy that indocyanine green (ICG), which is approved by the Pure Food and Drug Administration (FDA), is the only NIRF imaging probe that can be expected to the clinical detection of inflamed atherosclerotic plaques. Due to its lipophilicity, circulating ICG rapidly binds to low-density and high-density lipoproteins in the blood, following which this lipoprotein complex is absorbed by macrophages in the atherosclerotic plaque, internalizing the ICG. Rabbit models and human tissues *in vitro* have confirmed that plaque lipids, macrophages, and subendothelial deposits are targeted by ICG in fluorescence imaging (26). In addition, iron oxide nanoparticles (IONPs), which are another molecular prober, change the magnetic field through macrophage scavenger receptor–mediated endocytosis and are biocompatible and biodegradable in a wide range of applications (Figure 2) (20). Ikeda et al. designed activatable fluorescent probes equipped with highly compatible ICG and IONPs and observed a significant increase in the signal in the in the quantitative evaluation of NIRF. Moreover, in groups with different proportions of ICG, the mouse model of IONP-ICG20 showed a distinct NIRF signal reflecting the number of macrophages present (27).

Activated macrophages secrete proteolytic enzymes, including matrix metalloproteinases (MMPs) (Figure 1), which induce the discontinuation of fibrous caps and plaque destabilization. MMPs also overexpress cathepsin, which plays a key role in inflammation and interleukin (IL)-1β processing in atherosclerotic plaques (24). Knowledge of these proteases has allowed the development of “smart” probes specifically designed to identify when fluorescent signals emitted by macrophages switch from “off” to “on” under certain circumstances in fluorescence imaging. Narita et al. for instance, aimed to synthesize a fluorescent smart probe to detect specific fluorescence activation and image macrophages, and they encapsulated Peptide-ICG2 (optically silent under normal conditions; activates in the presence of the lysosomal enzyme) into phosphatidylserine-containing liposome (PICG2-PS-Lip) to achieve these requirements (Figure 2). When Peptide-ICG2 has been lysed with the lysosomal enzyme cathepsin B, which is highly expressed in the lysosomes of macrophages, the quenching effect of the peptide can be released, switching on ICG2 fluorescence in macrophages (18).

Although research on NIRF imaging probes is flourishing, poor NIRF penetration makes it far from clinical translation. The current trend is its combination with intravascular imaging to obtain morphological and molecular information on human coronary arteries (detailed later).

## Intravital Microscopy

Using the above fluorescent probes, a fluorescence microscopy technique named laser scanning IVM has also been used to detect macrophages (28). Through the use of laser sources and high-resolution microscopy, IVM with the stability of fluorescent proteins or probes was shown to enable real-time tracking of single or multiple macrophages in atherosclerosis *in vivo* (17, 21). A reliable tool for real-time visualization of macrophages allows the understanding of macrophage positional dynamics and how intravascular inflammation drives atherogenesis. Xiong et al. (29) used IVM to confirm that one of the reasons for attenuated atherosclerosis and monocyte/macrophage accumulation by vasostatin-2 is the blocking of chemotaxis and recruitment of inflammatory monocytes/macrophages. Furthermore, Williams et al. (30) performed a two-pronged approach macrophage dynamic model—using IVM to examine macrophage behavior in the living mouse and then added a long-term assessment of macrophage positioning by quantifying the location of stable phagocytic cargo carried by macrophages within plaques. IVM can also perform real-time imaging in live animals, but its operation process invasively exposes the location of atherosclerotic plaques, which may be more traumatic to experimental models, and is also difficult to transform into human models.

## Contrast-enhanced Ultrasound

This method utilizes acoustically active microbubbles to detect the endothelial-blood pool interface of the vascular compartment (31, 32). The ultrasound microbubbles of various inflammatory cells or inflammatory factors are introduced into the animal atherosclerotic model and targeted to combine with inflammatory cells or factors in the plaque, resulting in changes in the inflammatory response at the molecular level, which can be seen by local echo (33). Atkinson et al. (34) used CEU to identify and estimate the changes in macrophage burden that reflect the degree of progression in high-risk atherosclerosis in the evaluation of the therapeutic effect of anti-oxidant therapy. CEU could be used in the future as an early screening tool for potential atherosclerosis development. However, because of poor spatial localization and restricted to the vascular compartment, CEU is not an ideal method for detecting macrophages within atherosclerotic plaques.

In addition, sonodynamic therapy (SDT) is one of several new treatment methods that combine low-intensity ultrasound with sonosensitizers, which promotes direct macrophage reduction or macrophage apoptosis-induced endothelial cell apoptosis (35, 36). The combination of aminolaevulinic acid gold nanoparticles and SDT has noticeable advantages, including a high astuteness for pathological sites and low systemic toxicity, and may represent a promising alternative therapy (37). Overall, continued research shows that SDT is a novel treatment modality that can identify the optimal macrophage target in the treatment of atherosclerosis.

## Magnetic Resonance Imaging

MRI combines excellent spatial resolution with contrast of soft tissue morphology to semi-quantitatively detect macrophages (38–40). MRI delineates macrophage accumulation in atherosclerosis by combining nanoparticles represented by ultrasmall superparamagnetic iron oxide (USPIO) *in vivo* and gadolinium contrast. Macrophages engulf the ferromagnetic USPIO at the site of atherosclerosis and shorten the relaxation times of the surrounding water molecules due to the magnetic sensitivity of the USPIO. This can be seen on MRI imaging as signal loss in T2-weighted sequences (41, 42). USPIO has been used to identify plaque macrophages as a succedaneum of plaque inflammation in assessing atherosclerosis and setting risk stratification in human and animal models (43). Moreover, due to the slow absorption of USPIO, long-circulating times are required to procure an adequate accumulation to allow for MRI imaging. To address these practical and theoretical limitations, dual-targeted nanoparticles (NPs) equipped iron oxide NPs, and mito-magneto MRI contrast enhancement of the macrophage mitochondria were carried out to target the macrophages, and optimization of the composition of NPs was shown to achieve better recognition (44). Another dual-modal fluorescent iron oxide magnetic NP (MNP) method involves the use of folate-conjugated fluorescent dyed MNPs@OPE-PEG-NH2 to target the folate receptor, which is a marker of activated macrophages in which FR-β is specifically expressed (45). In addition, Tarin et al. (46) directed nanoparticles vectorized with gold coated iron oxide to CD163, the membrane receptor expressed by monocyte-macrophage lineage, as a potential strategy for the synthesis of targeted probes for macrophage imaging. Compared with USPIO, microparticles of iron oxide (MPIO) with a more significant MR contrast effect synthesized a dual-modal MPIO as a contrast agent act to render adhesion molecules and P-selectin on macrophages in the mouse model (47).

On the other hand, the key advantage of gadolinium contrast applied to MRI scanning lies in the enhancement of plaque tissue in dynamic kinetics, so that T1-weighted sequences can identify macrophages noninvasively. However, gadolinium contrast has obvious deficiencies, including a low relaxation rate, a short circulation time, rapid elimination by the kidney, and poor biocompatibility. Ongoing research may provide promising gadolinium contrast agents for MRI with both effective and targeted contrast abilities to enable macrophage detection (48, 49). Shen et al. (50) explored a novel lipopeptide nanoparticle, which contained gadolinium-based contrast agents and modified synthetic apolipoprotein A-I peptides. This new nanoparticle could significantly enhance the detection of plaque and reduce the adverse effects of gadolinium, and that the optimized spherical particles could further diminish adverse renal effects. Furthermore, Yu et al. (51) recently synthesized a gadolinium-doped oxide nanoparticle functionalized by hyaluronic acid (HA-GdIO NPs), which could be used for T1–T2 dual-modal contrast imaging of atherosclerosis through selective accumulation in CD44-overpressing macrophages, suggesting their potential as a contrast agent for the detection of macrophages.

It is necessary to optimize or develop new technologies to obtain more information on atherosclerotic plaques at a higher spatial resolution and to reduce significant imaging artifacts due to pulsatile vascular motion. The general demand for higher temporal and spatial resolution of vascular MRI may encourage the use of these higher field strengths. We expect that medical physics can solve the problem of the inhomogeneity of the magnetic field and transmission so that a magnetic field strength of more than 7 T can be transformed into clinical applications (52). Simultaneously, large-scale cohort and multicenter studies should perform more extensive scientific research for further clinical verification of novel multi-contrast sequences and molecular plaque imaging and to demonstrate their added value compared with standard techniques.

## Nuclear Imaging

**Figure 3:**
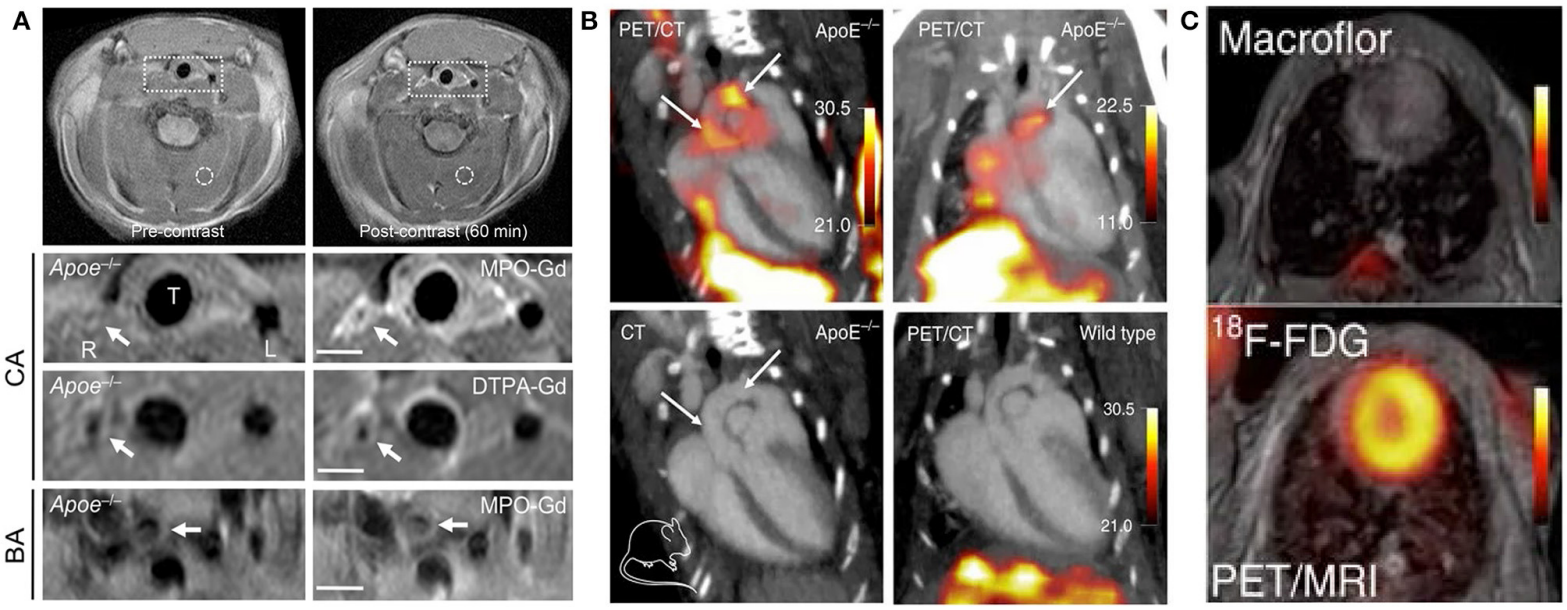
Examples of MRI and nuclear imaging for macrophages in atherosclerosis. **(A)** Pre- and post-MPO-Gd and DTPA-Gd T1-TSE imaging in TS Apoe^−/−^ mice. Representative as-acquired (2-cm field-of-view) MR images and higher-magnification images of unstable plaque (R), plaque-free artery (L) and stable plaque (BA) before and 60 min after probe administration, with corresponding time-course of CNR (MPO-Gd, filled symbols; DTPA-Gd, open symbols) in L (squares), R (circles), and BA (triangles) [adapted from (53)]. **(B)** Representative PET/CT images of several experiments in ApoE^−/−^ and wild-type control mice after IV Macroflor injection. **(C)** Cardiac PET images with respective agents (above: Macroflor, below: [18F] FDG) [adapted from (54)].

When inflammatory activation occurs, mononuclear phagocytic cells in plaque may alter their metabolic activity, making their detection by nuclear imaging possible. Nuclear technology involves the use of a radiotracer, which co-localizes with the target cell or receptor of interest in the plaque and emits gamma rays to probe the tracer, thereby displaying its functional characteristics. The main advantage to the widespread clinical application of nuclear imaging is its excellent sensitivity, which allows macrophages to be detected using a low tracer dose (25). Historically, SPECT and PET have been limited by their low spatial resolution; however, recent developments have seen functional information on molecular signals added to the anatomy data obtained by CT and MRI (Figure 3) (54). By developing novel tracers and nanoparticles that target macrophages with different hallmarks of plaque, radionuclide molecular imaging could provide new insights into the pathophysiology of atherosclerosis (55–57).

PET/CT combines the high sensitivity of PET, which offers biochemical function information, with the sectional anatomical detail provided by CT to reflect the signal of atherosclerotic blood vessel regions (58). 2-deoxy-2-[18F] fluoro-D-glucose ([18F] FDG) is a glucose analog that can be taken up by metabolically active tissues and its phosphorylation reaction can reflect the glucose metabolism of tissue cells. The uptake of [18F]FDG is directly proportional to the number of macrophages in high-risk plaques, which has been confirmed in histological studies with samples acquired by endarterectomy (59). So, PET/CT imaging of macrophages within carotid atherosclerosis plaque via [18F] FDG tracer has attracted significant attention from researchers. Other studies using [18F] FDG-PET/CT have confirmed that [18F] FDG uptake is significantly correlated with macrophage content (56, 59). To overcome the lack of specificity of the [18F] FDG tracer, both 3′-dexoy-3′[18F] fluorothymidine ([18F] FLT) and rHDL serving as markers of PET/CT imaging can target macrophage accumulation and activity in individuals with atherosclerosis (60, 61). Moreover, when the novel PET tracer ^64^Cu-DOTATATE was proposed and compared with [18F] FDG in the same animal, the PET signal emanating from atherosclerotic plaques was slightly higher for ^64^Cu-DOTATATE and persisted for longer, which was consistent with the alternatively activated macrophages (62).

Furthermore, PET/MRI and SPECT/MRI provide detection sensitivity and specificity an order of magnitude higher, thus requiring a lower concentration of nanoparticles compared to MRI. The intense radioactive signal detected by SPECT is focused on the identification and quantification of macrophages, and MRI is shown to improve focal localization and volume imaging in atherosclerosis. Recently, Cheng et al. (63) used SPECT/CT to design a multimodal probe specifically for apoptotic macrophages in vulnerable plaques by constructing a hybrid USPIO and PEG nanoparticle system and using Annexin V for targeting transport to areas with an abundance of apoptotic macrophages. Imaging revealed a clear signal in the macrophages with high uptake of the hybrid probe, which could identify higher-risk plaques and be helpful for volume determination with the precise lesion contour. A recent study showed that using ^64^Cu-ATSM as a PET/MRI imaging agent was beneficial for visualizing hypoxic macrophages in atherosclerotic animal models (64, 65). Subsequent studies translated nanobody-based radiotracer expressed on macrophages (^64^Cu-macrophage mannose receptor nanobody) to animal models and integrated it in a PET/MRI protocol that allowed evaluation of the macrophage burden and revealed several key features of atherosclerosis progression (56).

## Multimodality Imaging

Considerable effort has been made to combine the strengths of various imaging methods to better visualize macrophages. As multimodal imaging agents require target cells to take up a sufficient proportion of the contrast agent or nanoprobes to improve the sensitivity, researchers are going for creating multiple binding sites of contrast agents and compounding the hybrid targeted molecular probes. The broad range of multimodal imaging methods can be extended by performing imaging with several platforms, including PET/CT and PET/MRI. The contrast agent of CT and optical dual-modal imaging can maximize the capabilities of the high spatial resolution of CT and the high sensitivity of optical imaging, which has great potential in specifically targeting macrophages (66). Moreover, a dual-modal ultrasound/MRI contrast agent exploited by the Ji et al. observed macrophage enrichment in abdominal aortic atherosclerotic plaques. The synthesis and characterization of anti-CD68 receptor-targeted Fe-doped hollow silica nanoparticles (CD68-Fe-HSNs) was mainly composed of three parts: a CD68 receptor that was highly and specifically expressed on macrophage activation; HSNs with a stable shell and high biosafety as an excellent contrast agent for ultrasound imaging; and doped iron that provided T2-weighted MRI imaging (67).

The expression of the secreted biomarker osteopontin (OPN) is strongly associated with macrophage and foamy macrophage content, and plays a key role in plaque progression, including in the recruitment and viability of leukocytes and cytokines, and MMP expression. Qiao et al. (68) attached the OPN antibody to NaGdF4: Yb, Er@NaGdF4 up conversion nanoparticles covalently to construct a dual-modality imaging probe. Specific probe and upconversion optical imaging were then performed to visualize plaques induced by lowered and oscillatory shear stress in the carotid arteries of mice. In addition, the Li et al. (69) built ultrasound/optical dual-modality probe (Cy5.5-anti-OPN-PEG-PLA-PFOB, denoted as COP-NPs), which uses OPN targeted nanoparticles for the molecular imaging of foam macrophage cells, could be a promising tool for identifying the molecular characteristics of mice at high-risk of atherosclerosis.

The construction of well-designed, multi-modal nanoparticles not only facilitate imaging but may also temper both local and systemic immune cell inflammation. The specific accumulation of spherical polymeric nano constructs (SPNs) in lipid-rich plaques show nuclear imaging and optical imaging signals, and histological analysis confirms that SPNs are taken up by macrophages, indicating that it can accurately image them. Multifunctional, hybrid nanoparticles were reported to deliver the MTX system to macrophages to achieve an effective therapeutic strategy that inhibited atherosclerosis progression and potentially induced the absorption of vascular lesions (71). Using multimodal imaging techniques to evaluate drug capabilities also offers the potential for future clinical applications. Using multi-modal imaging techniques, Cecconi et al. (72) showed that colchicine could stabilize atherosclerotic plaques by reducing inflammatory activity and plaque burden while having no effect on macrophage immersion or plaque typology. The field of molecular imaging is growing, and it is anticipated that the increase in preclinical and clinical studies will accelerate the noninvasive, sensitive, and longitudinal assessment of macrophages in atherosclerosis.

## Optical Coherence Tomography

**Figure 4:**
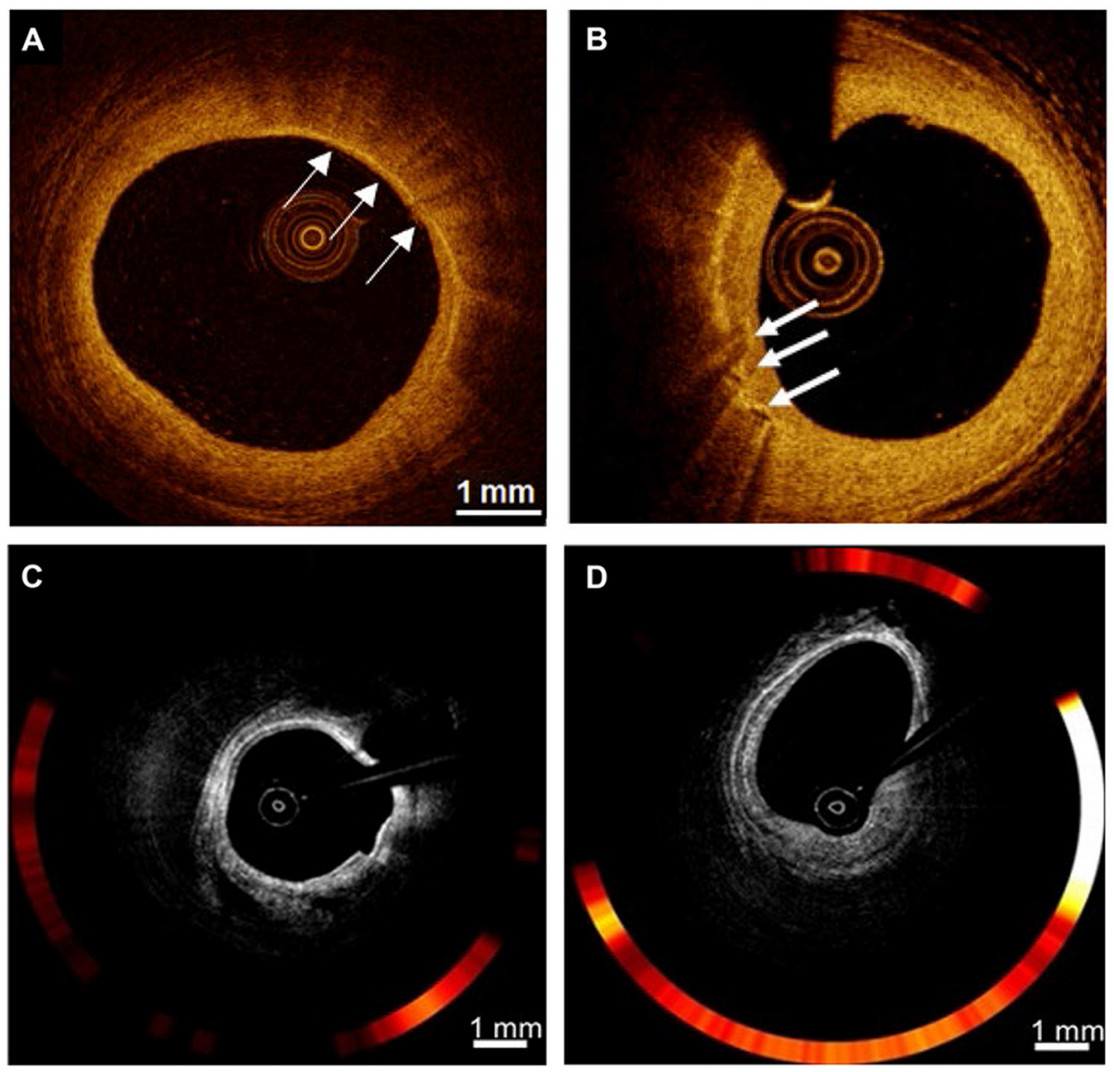
Examples of nonmolecular imaging for macrophages in atherosclerosis. **(A,B)** OCT cross-section images of the atherosclerotic vessel lumen, the location indicated by the white arrow is the macrophage. (**C,D)** Macrophages with robust NIRF signals on OCT-NIRF, and its content can be judged by red signal intensity; Focal plaque with surface infiltration of lipid-laden macrophages [adapted from (70)].

The most well-known form of nonmolecular imaging is optical coherence tomography (OCT), which measures the intensity of back-reflected infrared light. By producing high-resolution imaging (10 μm) in clinical real-time application or *in vivo*, OCT provides cross-sectional images of arterial tissue, including plaque characteristics, macrophages, and microchannels. Macrophage imaging is defined as when signals that exceed the intensity of background speckle noise are rich, distinct or convergent tufted areas are present (Figure 4) (73, 74), or when strongly linear images on the plaque surface accompanied by high attenuation (attenuation coefficient μt ≥ 10 mm^−1^) are seen (75). Multiple studies combined intravascular imaging with histology have targeted identification and quantification of macrophages present in coronary atherosclerotic plaques to reflect the capability of OCT. Using tissue property indexes to verify the accuracy of OCT in recognizing macrophages, Di Vito et al. then proposed a two-step algorithm for macrophage quantification. The algorithm first applied OCT-derived tissue property indexes, normalized standard deviation (NSD) with a cut-off value of 0.0570, then used a granulometry index to identify significant plaque inflammation with a sensitivity and specificity of 100 and 96.8%, respectively (76). With the extensive use of processing methods for automated OCT, the proposed NSD ratio method can accurately and quickly detect *in vivo* imaging of macrophage content within coronary atherosclerotic plaques simultaneously during standard OCT imaging system operation (77).

Macrophages are intrinsically linked to one of the indicators of atheroma progression and may also predict risk vulnerability (7). A study of inflammatory infiltration of ruptured plaques in ACS patients found a large macrophage burden, suggesting that plaque rupture might mainly be caused by chronic, low-grade background inflammation. In addition, a C-reactive protein value >3 mg/dL was found to be the only independent predictor of macrophage infiltration in the culprit plaque (78). In the CLIMA study of 1,003 patients who underwent coronary angiography and coronary artery OCT imaging, patients with macrophage inflammatory infiltrate had a higher risk of cardiac death and target vessel myocardial infarction (79). In the one-time acquisition of OCT images, we can obtain semi-quantitative images of macrophages as one of the main indicators in the evaluation of vulnerable plaques in clinical practice. However, because the image of macrophages is susceptible to artifacts and its interpretation is highly subjective, it is often necessary to combine other indicators when evaluating vulnerable plaques.

## OCT-NIRF

OCT reflects the morphological characteristics of atherosclerotic plaques but cannot directly provide information about their inflammatory activity. Intravascular NIRF, however, can image atherosclerosis at the molecular and cellular levels as well as inflammatory activity, although its vessel localization ability has limited clinical application. The recent development of an integrated imaging system using intravascular OCT-NIRF can achieve the precise co-localization of microstructural information and enzyme activity in atherosclerosis (Figure 4) (70, 80).

OCT-NIRF uses the FDA-approved contrast agent indocyanine green (ICG), which provides good imaging results for NIRF imaging and minimal renal toxicity during metabolism. Ughi et al. (81) proposed an automated algorithm that enabled full-automatic visualization of dual-modal OCT-NIRF pullbacks, and provided accurate and effective calibration of NIRF data for quantifying molecular conditions in atherosclerotic vessel walls, thus greatly increasing the application of this technology. Lee et al. (82) also demonstrated the feasibility of integrated OCT-NIRF structural molecular imaging by identifying lipid-rich inflammatory atherosclerosis and concluded that the dual-mode imaging method had an enhanced ability to detect high-risk plaques. OCT-NIRF has also been shown to be effective for imaging high-risk plaques, and can safely and efficiently perform dual-pattern microstructures and coronary artery fluorescence imaging in humans (26, 83). Additionally, high-risk plaques with intraplaque hemorrhage and heme degradation products can be detected and monitored by near-infrared autofluorescence, which is a novel technology that reflects plaque instability, as seen in human carotid endarterectomy samples (84).

In addition to OCT, intraluminal imaging tomography also includes intravascular ultrasonography (IVUS) and near-infrared spectroscopy (NIRS), although these methods cannot independently identify macrophages (85–87). A small sample study showed that CD163-positive macrophage infiltration could be predicted if positive remodeling and a large necrotic core without calcification were seen on virtual histology IVUS imaging (88); however, this result was not supported by another research. While IVUS-NIRS imaging seems to provide effective solutions for the visual diagnosis and quantitative analysis of lipid plaques, a study of the consistency of IVUS-NIRS and OCT for lipid pool detection showed that the false positive and false negative rates were higher with IVUS-NIRS imaging. Macrophage clusters were observed in most false-positive cases for lipid detection, and the presence of different types of calcification was seen to be more common in false-negative cases. The results of that study revealed that IVUS-NIRS was less capable of identifying macrophages because the presence of calcium components in plaques affected the imaging of lipids (89).

Because intravascular imaging is increasingly being used in PCI, its only difference from NIRF-OCT or NIRF-IVUS is that the targeted molecular imaging agent is injected intravenously at the beginning of PCI, which does not increase the burden of clinical operations. Intravascular imaging combined with molecular imaging technology is based on the key driving factors of coronary events, such as inflammatory macrophages, offering a new dimension for the risk assessment of atherosclerotic plaques. The new generational atherosclerosis score to be established can integrate coronary vascular morphological and molecular characteristics, which reflects the pathophysiological process of the culprit and nonculprit arteries. Furthermore, the molecular structural atheroma score will identify high-risk lesions, arteries, and patients, allowing the ability to personalize medical therapy to those at the highest risk.

## Conclusion

Atherosclerotic plaque vulnerability and progression, which are reflected by macrophages, represent one of the principal risk-factors for acute cardiovascular events. This raises the importance of exploring new detection methods and treatments to protect the coronary arteries. An important feature of molecular imaging is that it is noninvasive, which makes it an attractive method to consider for use in widespread screening. With its extremely high sensitivity and specificity, nonmolecular imaging can qualitatively and quantitatively analyze macrophages, and it has made an important contribution to the development of precise treatment plans for individual high-risk patients. We will continue to meet challenges as the questions underlying the clinical application of imaging push the limits of our technologies. The development of molecular and nonmolecular imaging will greatly improve our ability to diagnose atherosclerosis at an early stage, facilitating early intervention and the initiation of individualized therapy.

## Author Contributions

ZL designed and wrote the review and supervised and critically reviewed the complete manuscript. HT performed the literature search and prepared the figures. YT performed revisions and critically discussed the completed manuscript. All authors read and approved the final manuscript.

## Conflict of Interest

The authors declare that the research was conducted in the absence of any commercial or financial relationships that could be construed as a potential conflict of interest.

## References

[1] Joh JH Cho S. Cardiovascular risk of carotid atherosclerosis: global consensus beyond societal guidelines. Lancet Glob Health. (2020) 8:e625–e6. 10.1016/S2214-109X(20)30132-7 32353304

[2] Timmis A Townsend N Gale CP Torbica A Lettino M Petersen SE. European Society of Cardiology: cardiovascular disease statistics 2019. Eur Heart J. (2020) 41:12–85. 10.1093/eurheartj/ehz859 31820000

[3] Chattopadhyay A Kwartler CS Kaw K Li Y Kaw A Chen J. Cholesterol-induced phenotypic modulation of smooth muscle cells to macrophage/fibroblast-like cells is driven by an unfolded protein response. Arterioscler Thromb Vasc Biol. (2021) 41:302–16. 10.1161/ATVBAHA.120.315164 33028096 PMC7752246

[4] Yan J Horng T. Lipid metabolism in regulation of macrophage functions. Trends Cell Biol. (2020) 30:979–89. 10.1016/j.tcb.2020.09.006 33036870

[5] Lightbody RJ Taylor JMW Dempsie Y Graham A. MicroRNA sequences modulating inflammation and lipid accumulation in macrophage “foam” cells: implications for atherosclerosis. World J Cardiol. (2020) 12:303–33. 10.4330/wjc.v12.i7.303 32843934 PMC7415235

[6] Knuuti J Wijns W Saraste A Capodanno D Barbato E Funck-Brentano C. 2019 ESC Guidelines for the diagnosis and management of chronic coronary syndromes. Eur Heart J. (2020) 41:407–77. 10.1093/eurheartj/ehz425 31504439

[7] Eligini S Cosentino N Fiorelli S Fabbiocchi F Niccoli G Refaat H. Biological profile of monocyte-derived macrophages in coronary heart disease patients: implications for plaque morphology. Sci Rep. (2019) 9:8680. 10.1038/s41598-019-44847-3 31213640 PMC6581961

[8] Gonzalez L Trigatti BL. Macrophage apoptosis and necrotic core development in atherosclerosis: a rapidly advancing field with clinical relevance to imaging and therapy. Can J Cardiol. (2017) 33:303–12. 10.1016/j.cjca.2016.12.010 28232016

[9] Chen W Zhang F Ju Y Hong J Ding Y. Gold nanomaterial engineering for macrophage-mediated inflammation and tumor treatment. Adv Healthc Mater. (2020) 10:e2000818. 10.1002/adhm.202000818 33128505

[10] MacRitchie N Grassia G Noonan J Garside P Graham D Maffia P. Molecular imaging of atherosclerosis: spotlight on Raman spectroscopy and surface-enhanced Raman scattering. Heart. (2018) 104:460–7. 10.1136/heartjnl-2017-311447 29061690 PMC5861389

[11] Zong C Xu M Xu LJ Wei T Ma X Zheng XS. Surface-Enhanced Raman Spectroscopy for Bioanalysis: reliability and Challenges. Chem Rev. (2018) 118:4946–80. 10.1021/acs.chemrev.7b00668 29638112

[12] Matthaus C Krafft C Dietzek B Brehm BR Lorkowski S Popp J. Noninvasive imaging of intracellular lipid metabolism in macrophages by Raman microscopy in combination with stable isotopic labeling. Anal Chem. (2012) 84:8549–56. 10.1021/ac3012347 22954250

[13] Stiebing C Meyer T Rimke I Matthaus C Schmitt M Lorkowski S. Real-time Raman and SRS imaging of living human macrophages reveals cell-to-cell heterogeneity and dynamics of lipid uptake. J Biophotonics. (2017) 10:1217–26. 10.1002/jbio.201600279 28164480

[14] Pissuwan D Hattori Y. Detection of adhesion molecules on inflamed macrophages at early-stage using SERS probe gold nanorods. Nanomicro Lett. (2017) 9:8. 10.1007/s40820-016-0111-7 30460305 PMC6223776

[15] Ye S Wheeler MC McLaughlan JR Tamang A Diggle CP Cespedes O. Developing hollow-channel gold nanoflowers as trimodal intracellular nanoprobes. Int J Mol Sci. (2018) 19:2327. 10.3390/ijms19082327 30096801 PMC6121537

[16] Leppanen O Ekstrand M Brasen JH Levin M. Bioluminescence imaging of energy depletion in vascular pathology: patent ductus arteriosus and atherosclerosis. J Biophotonics. (2012) 5:336–44. 10.1002/jbio.201100096 22134948

[17] McArdle S Buscher K Ghosheh Y Pramod AB Miller J Winkels H. Migratory and dancing macrophage subsets in atherosclerotic lesions. Circ Res. (2019) 125:1038–51. 10.1161/CIRCRESAHA.119.315175 31594470 PMC7201888

[18] Narita Y Shimizu K Ikemoto K Uchino R Kosugi M Maess MB. Macrophage-targeted, enzyme-triggered fluorescence switch-on system for detection of embolism-vulnerable atherosclerotic plaques. J Control Release. (2019) 302:105–15. 10.1016/j.jconrel.2019.03.025 30936020

[19] Abd-Elrahman I Meir K Kosuge H Ben-Nun Y Weiss Sadan T Rubinstein C. Characterizing cathepsin activity and macrophage subtypes in excised human carotid plaques. Stroke. (2016) 47:1101–8. 10.1161/STROKEAHA.115.011573 26941255

[20] Kim M Sahu A Kim GB Nam GH Um W Shin SJ. Comparison of *in vivo* targeting ability between cRGD and collagen-targeting peptide conjugated nano-carriers for atherosclerosis. J Control Release. (2018) 269:337–46. 10.1016/j.jconrel.2017.11.033 29175140

[21] Li Y Liu TM. Discovering macrophage functions using *in vivo* optical imaging techniques. Front Immunol. (2018) 9:502. 10.3389/fimmu.2018.00502 29599778 PMC5863475

[22] Pajarinen J Lin TH Sato T Loi F Yao Z Konttinen YT. Establishment of green fluorescent protein and firefly luciferase expressing mouse primary macrophages for *in vivo* bioluminescence imaging. PLoS One. (2015) 10:e0142736. 10.1371/journal.pone.0142736 26555613 PMC4640705

[23] Deng H Konopka CJ Cross TL Swanson KS Dobrucki LW Smith AM. Multimodal nanocarrier probes reveal superior biodistribution quantification by isotopic analysis over fluorescence. ACS Nano. (2020) 14:509–23. 10.1021/acsnano.9b06504 31887006 PMC7377915

[24] Birch GP Campbell T Bradley M Dhaliwal K. Optical molecular imaging of inflammatory cells in interventional medicine-an emerging strategy. Front Oncol. (2019) 9:882. 10.3389/fonc.2019.00882 31572676 PMC6751259

[25] Celeng C de Keizer B Merkely B de Jong P Leiner T Takx RAP. PET molecular targets and near-infrared fluorescence imaging of atherosclerosis. Curr Cardiol Rep. (2018) 20:11. 10.1007/s11886-018-0953-3 29435774 PMC5809554

[26] Verjans JW Osborn EA Ughi GJ Calfon Press MA Hamidi E Antoniadis AP. Targeted near-infrared fluorescence imaging of atherosclerosis: clinical and intracoronary evaluation of indocyanine green. JACC Cardiovasc Imaging. (2016) 9:1087–95. 10.1016/j.jcmg.2016.01.034 27544892 PMC5136528

[27] Ikeda H Ishii A Sano K Chihara H Arai D Abekura Y. Activatable fluorescence imaging of macrophages in atherosclerotic plaques using iron oxide nanoparticles conjugated with indocyanine green. Atherosclerosis. (2018) 275:1–10. 10.1016/j.atherosclerosis.2018.05.028 29852399

[28] Choi M Kwok SJ Yun SH. *In vivo* fluorescence microscopy: lessons from observing cell behavior in their native environment. Physiology (Bethesda). (2015) 30:40–9. 10.1152/physiol.00019.2014 25559154 PMC4285577

[29] Xiong W Wang X Dai D Zhang B Lu L Tao R. The anti-inflammatory vasostatin-2 attenuates atherosclerosis in ApoE-/- mice and inhibits monocyte/macrophage recruitment. Thromb Haemost. (2017) 117:401–14. 10.1160/TH16-06-0475 27831589

[30] Williams JW Martel C Potteaux S Esaulova E Ingersoll MA Elvington A. Limited macrophage positional dynamics in progressing or regressing murine atherosclerotic plaques-brief report. Arterioscler Thromb Vasc Biol. (2018) 38:1702–10. 10.1161/ATVBAHA.118.311319 29903736 PMC6202234

[31] Kaufmann BA Wei K Lindner JR. Contrast echocardiography. Curr Probl Cardiol. (2007) 32:51–96. 10.1016/j.cpcardiol.2006.10.004 17208647

[32] Perrone-Filardi P Dellegrottaglie S Rudd JH Costanzo P Marciano C Vassallo E. Molecular imaging of atherosclerosis in translational medicine. Eur J Nucl Med Mol Imaging. (2011) 38:969–75. 10.1007/s00259-010-1697-5 21174089

[33] Brown E Lindner JR. Ultrasound molecular imaging: principles and applications in cardiovascular medicine. Curr Cardiol Rep. (2019) 21:30. 10.1007/s11886-019-1117-9 30887129

[34] Atkinson T Packwood W Xie A Liang S Qi Y Ruggeri Z. Assessment of novel antioxidant therapy in atherosclerosis by contrast ultrasound molecular imaging. J Am Soc Echocardiogr. (2018) 31:1252–9.e1. 10.1016/j.echo.2018.07.017 30213420 PMC6218294

[35] Geng C Zhang Y Hidru TH Zhi L Tao M Zou L. Sonodynamic therapy: a potential treatment for atherosclerosis. Life Sci. (2018) 207:304–13. 10.1016/j.lfs.2018.06.018 29940244

[36] Yao J Gao W Wang Y Wang L Diabakte K Li J. Sonodynamic therapy suppresses neovascularization in atherosclerotic plaques via macrophage apoptosis-induced endothelial cell apoptosis. JACC Basic Transl Sci. (2020) 5:53–65. 10.1016/j.jacbts.2019.10.007 32043020 PMC7000870

[37] Goncalvez KO Vieira DP Courrol LC. Study of THP-1 macrophage viability after sonodynamic therapy using methyl ester of 5-aminolevulinic acid gold nanoparticles. Ultrasound Med Biol. (2018) 44:2009–17. 10.1016/j.ultrasmedbio.2018.05.012 29936026

[38] Swirski FK Nahrendorf M. Imaging macrophage development and fate in atherosclerosis and myocardial infarction. Immunol Cell Biol. (2013) 91:297–303. 10.1038/icb.2012.72 23207281 PMC3875224

[39] Bakerman I Wardak M Nguyen PK. Molecular imaging of inflammation in ischemic heart disease. Curr Cardiovasc Imaging Rep. (2018) 11:13. 10.1007/s12410-018-9454-4 31186825 PMC6559744

[40] Wust RCI Calcagno C Daal MRR Nederveen AJ Coolen BF Strijkers GJ. Emerging magnetic resonance imaging techniques for atherosclerosis imaging. Arterioscler Thromb Vasc Biol. (2019) 39:841–49. 10.1161/ATVBAHA.118.311756 30917678

[41] Sadat U Usman A Gillard JH. Imaging pathobiology of carotid atherosclerosis with ultrasmall superparamagnetic particles of iron oxide: an update. Curr Opin Cardiol. (2017) 32:437–40. 10.1097/HCO.0000000000000413 28463893 PMC5617556

[42] Hu ZP Fang XL Sheng B Guo Y Yu YQ. Melatonin inhibits macrophage infiltration and promotes plaque stabilization by upregulating anti-inflammatory HGF/c-Met system in the atherosclerotic rabbit: USPIO-enhanced MRI assessment. Vascul Pharmacol. (2020) 127:106659. 10.1016/j.vph.2020.106659 32068091

[43] Merinopoulos I Gunawardena T Stirrat C Cameron D Eccleshall SC Dweck MR. Diagnostic applications of ultrasmall superparamagnetic particles of iron oxide for imaging myocardial and vascular inflammation. JACC Cardiovasc Imaging. (2020). 10.1016/j.jcmg.2020.06.038. [Epub ahead of print]. 32861658

[44] Banik B Surnar B Askins BW Banerjee M Dhar S. Dual-targeted synthetic nanoparticles for cardiovascular diseases. ACS Appl Mater Interfaces. (2020) 12:6852–62. 10.1021/acsami.9b19036 31886643

[45] Yao Y Li B Yin C Cong F Ma GS Liu NF. A folate-conjugated dual-modal fluorescent magnetic resonance imaging contrast agent that targets activated macrophages *in vitro* and *in vivo*. J Biomed Nanotechnol. (2016) 12:2161–71. 10.1166/jbn.2016.2316 29372808

[46] Tarin C Carril M Martin-Ventura JL Markuerkiaga I Padro D Llamas-Granda P. Targeted gold-coated iron oxide nanoparticles for CD163 detection in atherosclerosis by MRI. Sci Rep. (2015) 5:17135. 10.1038/srep17135 26616677 PMC4663748

[47] Mucherah W Thomas K. Reducing barriers to primary school education for girls in rural Kenya: reusable pads' intervention. Int J Adolesc Med Health. (2017) 31:20170005. 10.1515/ijamh-2017-0005 28628478

[48] Sigalov AB. Nature-inspired nanoformulations for contrast-enhanced *in vivo* MR imaging of macrophages. Contrast Media Mol Imaging. (2014) 9:372–82. 10.1002/cmmi.1587 24729189 PMC4197124

[49] Nguyen TH Bryant H Shapsa A Street H Mani V Fayad ZA. Manganese G8 dendrimers targeted to oxidation-specific epitopes: *in vivo* MR imaging of atherosclerosis. J Magn Reson Imaging. (2015) 41:797–805. 10.1002/jmri.24606 24610640 PMC4160426

[50] Shen ZT Zheng S Gounis MJ Sigalov AB. Diagnostic magnetic resonance imaging of atherosclerosis in apolipoprotein E knockout mouse model using macrophage-targeted gadolinium-containing synthetic lipopeptide nanoparticles. PLoS One. (2015) 10:e0143453. 10.1371/journal.pone.0143453 26569115 PMC4646679

[51] Yu M Niu Y Zhou D Jiang R Zhang L Ju H. Hyaluronic acid-functionalized gadolinium doped iron oxide nanoparticles for atherosclerosis-targeted Mr imaging. J Biomed Nanotechnol. (2019) 15:127–37. 10.1166/jbn.2019.2660 30480520

[52] Erturk MA Wu X Eryaman Y Van de Moortele PF Auerbach EJ Lagore RL. Toward imaging the body at 10.5 tesla. Magn Reson Med. (2017) 77:434–43. 10.1002/mrm.26487 27770469 PMC5191924

[53] Rashid I Maghzal GJ Chen YC Cheng D Talib J Newington D. Myeloperoxidase is a potential molecular imaging and therapeutic target for the identification and stabilization of high-risk atherosclerotic plaque. Eur Heart J. (2018) 39:3301–10. 10.1093/eurheartj/ehy419 30219874

[54] Keliher EJ Ye YX Wojtkiewicz GR Aguirre AD Tricot B Senders ML. Polyglucose nanoparticles with renal elimination and macrophage avidity facilitate PET imaging in ischaemic heart disease. Nat Commun. (2017) 8:14064. 10.1038/ncomms14064 28091604 PMC5241815

[55] Senders ML Que X Cho YS Yeang C Groenen H Fay F. PET/MR imaging of malondialdehyde-acetaldehyde epitopes with a human antibody detects clinically relevant atherothrombosis. J Am Coll Cardiol. (2018) 71:321–35. 10.1016/j.jacc.2017.11.036 29348025 PMC5995462

[56] Senders ML Hernot S Carlucci G van de Voort JC Fay F Calcagno C. Nanobody-facilitated multiparametric PET/MRI phenotyping of atherosclerosis. JACC Cardiovasc Imaging. (2019) 12:2015–26. 10.1016/j.jcmg.2018.07.027 30343086 PMC6461528

[57] Sriranjan RS Tarkin JM Evans NR Le EPV Chowdhury MM Rudd JHF. Atherosclerosis imaging using PET: insights and applications. Br J Pharmacol. (2019). 10.1111/bph.14868. [Epub ahead of print]. 31517992

[58] Lairez O Hyafil F. A clinical role of PET in atherosclerosis and vulnerable plaques? Semin Nucl Med. (2020) 50:311–18. 10.1053/j.semnuclmed.2020.02.017 32540028

[59] Piri R Gerke O Hoilund-Carlsen PF. Molecular imaging of carotid artery atherosclerosis with PET: a systematic review. Eur J Nucl Med Mol Imaging. (2020) 47:2016–25. 10.1007/s00259-019-04622-y 31786626

[60] Salzsieder E Berg S. Accuracy evaluation of a CE-Marked system for self-monitoring of blood glucose with three reagent system lots following ISO 15197:2013. J Diabetes Sci Technol. (2015) 10:238–9. 10.1177/1932296815606471 26394775 PMC4738220

[61] Perez-Medina C Binderup T Lobatto ME Tang J Calcagno C Giesen L. *In vivo* PET imaging of HDL in multiple atherosclerosis models. JACC Cardiovasc Imaging. (2016) 9:950–61. 10.1016/j.jcmg.2016.01.020 27236528 PMC5589956

[62] Pedersen SF Sandholt BV Keller SH Hansen AE Clemmensen AE Sillesen H. 64Cu-DOTATATE PET/MRI for detection of activated macrophages in carotid atherosclerotic plaques: studies in patients undergoing endarterectomy. Arterioscler Thromb Vasc Biol. (2015) 35:1696–703. 10.1161/ATVBAHA.114.305067 25977567 PMC4479665

[63] Cheng D Li X Zhang C Tan H Wang C Pang L. Detection of vulnerable atherosclerosis plaques with a dual-modal single-photon-emission computed tomography/magnetic resonance imaging probe targeting apoptotic macrophages. ACS Appl Mater Interfaces. (2015) 7:2847–55. 10.1021/am508118x 25569777

[64] Nie X Laforest R Elvington A Randolph GJ Zheng J Voller T. PET/MRI of hypoxic atherosclerosis using 64Cu-ATSM in a rabbit model. J Nucl Med. (2016) 57:2006–11. 10.2967/jnumed.116.172544 27390157 PMC5126698

[65] Nie X Elvington A Laforest R Zheng J Voller TF Zayed MA. (64)Cu-ATSM positron emission tomography/magnetic resonance imaging of hypoxia in human atherosclerosis. Circ Cardiovasc Imaging. (2020) 13:e009791. 10.1161/CIRCIMAGING.119.009791 31910670 PMC7328725

[66] Ding J Wang Y Ma M Zhang Y Lu S Jiang Y. CT/fluorescence dual-modal nanoemulsion platform for investigating atherosclerotic plaques. Biomaterials. (2013) 34:209–16. 10.1016/j.biomaterials.2012.09.025 23069709

[67] Ji R Li X Zhou C Tian Q Li C Xia S. Identifying macrophage enrichment in atherosclerotic plaques by targeting dual-modal US imaging/MRI based on biodegradable Fe-doped hollow silica nanospheres conjugated with anti-CD68 antibody. Nanoscale. (2018) 10:20246–55. 10.1039/C8NR04703K 30361722

[68] Qiao R Qiao H Zhang Y Wang Y Chi C Tian J. Molecular imaging of vulnerable atherosclerotic plaques *in vivo* with osteopontin-specific upconversion nanoprobes. ACS Nano. (2017) 11:1816–25. 10.1021/acsnano.6b07842 28121134

[69] Li S Gou T Wang Q Chen M Chen Z Xu M. Ultrasound/optical dual-modality imaging for evaluation of vulnerable atherosclerotic plaques with osteopontin targeted nanoparticles. Macromol Biosci. (2020) 20:e1900279. 10.1002/mabi.201900279 31885210

[70] Kim S Lee MW Kim TS Song JW Nam HS Cho HS. Intracoronary dual-modal optical coherence tomography-near-infrared fluorescence structural-molecular imaging with a clinical dose of indocyanine green for the assessment of high-risk plaques and stent-associated inflammation in a beating coronary artery. Eur Heart J. (2016) 37:2833–44. 10.1093/eurheartj/ehv726 26787442

[71] Stigliano C Ramirez MR Singh JV Aryal S Key J Blanco E. Methotraxate-loaded hybrid nanoconstructs target vascular lesions and inhibit atherosclerosis progression in ApoE^−/−^ mice. Adv Healthc Mater. (2017) 6:1601286. 10.1002/adhm.201601286 28402587

[72] Cecconi A Vilchez-Tschischke JP Mateo J Sanchez-Gonzalez J Espana S Fernandez-Jimenez R. Effects of colchicine on atherosclerotic plaque stabilization: a multimodality imaging study in an animal model. J Cardiovasc Transl Res. (2020) 14:150–60. 10.1007/s12265-020-09974-7 32140929

[73] Tearney GJ. OCT imaging of macrophages: a bright spot in the study of inflammation in human atherosclerosis. JACC Cardiovasc Imaging. (2015) 8:73–75. 10.1016/j.jcmg.2014.09.019 25592697

[74] Di Vito L Yoon JH Kato K Yonetsu T Vergallo R Costa M. Comprehensive overview of definitions for optical coherence tomography-based plaque and stent analyses. Coron Artery Dis. (2014) 25:172–85. 10.1097/MCA.0000000000000072 24356250

[75] Uemura S Ishigami K Soeda T Okayama S Sung JH Nakagawa H. Thin-cap fibroatheroma and microchannel findings in optical coherence tomography correlate with subsequent progression of coronary atheromatous plaques. Eur Heart J. (2012) 33:78–85. 10.1093/eurheartj/ehr284 21831910

[76] Di Vito L Agozzino M Marco V Ricciardi A Concardi M Romagnoli E. Identification and quantification of macrophage presence in coronary atherosclerotic plaques by optical coherence tomography. Eur Heart J Cardiovasc Imaging. (2015) 16:807–13. 10.1093/ehjci/jeu307 25588802

[77] Wanninayake US Subedi B Fitzpatrick PF. pH and deuterium isotope effects on the reaction of trimethylamine dehydrogenase with dimethylamine. Arch Biochem Biophys. (2019) 676:108136. 10.1016/j.abb.2019.108136 31604072 PMC6924622

[78] Scalone G Niccoli G Refaat H Vergallo R Porto I Leone AM. Not all plaque ruptures are born equal: an optical coherence tomography study. Eur Heart J Cardiovasc Imaging. (2017) 18:1271–77. 10.1093/ehjci/jew208 28013285

[79] Romagnoli E Gatto L Prati F. The CLIMA study: assessing the risk of myocardial infarction with a new anatomical score. Eur Heart J Suppl. (2019) 21:B80–B3. 10.1093/eurheartj/suz032 30948958 PMC6439910

[80] Khraishah H Jaffer FA. Intravascular molecular imaging: near-infrared fluorescence as a new frontier. Front Cardiovasc Med. (2020) 7:587100. 10.3389/fcvm.2020.587100 33330648 PMC7719823

[81] Ughi GJ Verjans J Fard AM Wang H Osborn E Hara T. Dual modality intravascular optical coherence tomography (OCT) and near-infrared fluorescence (NIRF) imaging: a fully automated algorithm for the distance-calibration of NIRF signal intensity for quantitative molecular imaging. Int J Cardiovasc Imaging. (2015) 31:259–68. 10.1007/s10554-014-0556-z 25341407 PMC4344893

[82] Lee S Lee MW Cho HS Song JW Nam HS Oh DJ. Fully integrated high-speed intravascular optical coherence tomography/near-infrared fluorescence structural/molecular imaging *in vivo* using a clinically available near-infrared fluorescence-emitting indocyanine green to detect inflamed lipid-rich atheromata in coronary-sized vessels. Circ Cardiovasc Interv. (2014) 7:560–9. 10.1161/CIRCINTERVENTIONS.114.001498 25074255

[83] Ughi GJ Wang H Gerbaud E Gardecki JA Fard AM Hamidi E. Clinical characterization of coronary atherosclerosis with dual-modality OCT and near-infrared autofluorescence imaging. JACC Cardiovasc Imaging. (2016) 9:1304–14. 10.1016/j.jcmg.2015.11.020 26971006 PMC5010789

[84] Htun NM Chen YC Lim B Schiller T Maghzal GJ Huang AL. Near-infrared autofluorescence induced by intraplaque hemorrhage and heme degradation as marker for high-risk atherosclerotic plaques. Nat Commun. (2017) 8:75. 10.1038/s41467-017-00138-x 28706202 PMC5509677

[85] Mintz GS. Clinical utility of intravascular imaging and physiology in coronary artery disease. J Am Coll Cardiol. (2014) 64:207–22. 10.1016/j.jacc.2014.01.015 24530669

[86] Waksman R Di Mario C Torguson R Ali ZA Singh V Skinner WH. Identification of patients and plaques vulnerable to future coronary events with near-infrared spectroscopy intravascular ultrasound imaging: a prospective, cohort study. Lancet. (2019) 394:1629–37. 10.1016/S0140-6736(19)31794-5 31570255

[87] Johnson TW Raber L di Mario C Bourantas C Jia H Mattesini A. Clinical use of intracoronary imaging. Part 2: acute coronary syndromes, ambiguous coronary angiography findings, and guiding interventional decision-making: an expert consensus document of the European Association of Percutaneous Cardiovascular Interventions. Eur Heart J. (2019) 40:2566–84. 10.1093/eurheartj/ehz332 31112213

[88] Sato T Kameyama T Ueno H Inoue H. Intravascular ultrasound predictors of CD163 positive macrophage infiltration. J Interv Cardiol. (2014) 27:317–24. 10.1111/joic.12111 24612144

[89] Di Vito L Imola F Gatto L Romagnoli E Limbruno U Marco V. Limitations of OCT in identifying and quantifying lipid components: an *in vivo* comparison study with IVUS-NIRS. EuroIntervention. (2017) 13:303–11. 10.4244/EIJ-D-16-00317 27973332

